# Fabrication of Curcumin Diethyl γ-Aminobutyrate-Loaded Chitosan-Coated Magnetic Nanocarriers for Improvement of Cytotoxicity against Breast Cancer Cells

**DOI:** 10.3390/polym14245563

**Published:** 2022-12-19

**Authors:** Supakarn Hansapaiboon, Bryan Paul Bulatao, Feuangthit Niyamissara Sorasitthiyanukarn, Pongsakorn Jantaratana, Nonthaneth Nalinratana, Opa Vajragupta, Pranee Rojsitthisak, Pornchai Rojsitthisak

**Affiliations:** 1 Center of Excellence in Natural Products for Ageing and Chronic Diseases, Chulalongkorn University, Bangkok 10330, Thailand; 2 Pharmaceutical Sciences and Technology Program, Faculty of Pharmaceutical Sciences, Chulalongkorn University, Bangkok 10330, Thailand; 3 Department of Industrial Pharmacy, College of Pharmacy, University of the Philippines Manila, Manila 1000, Philippines; 4 Metallurgy and Materials Science Research Institute, Chulalongkorn University, Bangkok 10330, Thailand; 5 Department of Physics, Faculty of Science, Kasetsart University, Bangkok 10900, Thailand; 6 Department of Pharmacology and Physiology, Faculty of Pharmaceutical Sciences, Chulalongkorn University, Bangkok 10330, Thailand; 7 Molecular Probes for Imaging Research Network, Faculty of Pharmaceutical Sciences, Chulalongkorn University, Bangkok 10330, Thailand; 8 Department of Food and Pharmaceutical Chemistry, Faculty of Pharmaceutical Sciences, Chulalongkorn University, Bangkok 10330, Thailand

**Keywords:** iron oxide nanoparticles, magnetic targeting delivery, prodrug, Box–Behnken design, cytotoxicity

## Abstract

This study shows the effectiveness of magnetic-guide targeting in the delivery of curcumin diethyl γ-aminobutyrate (CUR-2GE), a prodrug of curcumin (CUR) previously synthesized to overcome unfavorable physicochemical properties of CUR. In this study, chitosan (Ch)-coated iron oxide nanoparticles (Ch-IONPs) were fabricated and optimized using Box–Behnken design-based response surface methodology for delivery of CUR-2GE. Ch was used as a coating material on the nanoparticle surface to avoid aggregation. The optimized condition for preparing Ch-IONPs consisted of using 4 mg Ch fabricated at pH 11 under a reaction temperature of 85 °C. The optimized Ch-IONPs were successfully loaded with CUR-2GE with sufficient loading capacity (1.72 ± 0.01%) and encapsulation efficiency (94.9 ± 0.8%). The obtained CUR-2GE-loaded Ch-IONPs (CUR-2GE-Ch-IONPs) exhibited desirable characteristics including a particle size of less than 50 nm based on TEM images, superparamagnetic property, highly crystalline IONP core, sufficient stability, and sustained-release profile. In the presence of permanent magnets, CUR-2GE-Ch-IONPs significantly increased cellular uptake and cytotoxicity toward MDA-MB-231 with a 12-fold increase in potency compared to free CUR-2GE, indicating the potential of magnetic-field assisted delivery of CUR-2GE-Ch-IONPs for the treatment of triple-negative breast cancer.

## 1. Introduction

Magnetic nanoparticles, commonly made of iron oxide nanoparticles (IONPs), have attracted some attention as a treatment option for cancer due to their multifunctional properties, biocompatibility, and low toxicity in the human body [1]. Moreover, IONPs can be controlled by a magnetic force applied to a target site and can decrease the distribution of drugs, thus reducing the unwanted effects of drugs on healthy cells [2]. However, uncoated IONPs easily aggregate and are recognized by the immune cells, resulting in rapid elimination from the body [3]. A surface coating has been used to enhance the blood circulation time of the IONPs. Several studies have employed polymers due to their biocompatible, biodegradable, and non-toxic properties [4]. In addition, the synthesis of IONPs has some critical conditions that must be optimally controlled, such as particle size, shape, surface coating, etc., because they facilitate diffusion, increase the accumulation of IONPs at the targeted site, and prevent the IONPs from being easily eliminated from the body system [5,6,7,8].

Among the commonly used types of iron oxide, including hematite, maghemite, and magnetite, the latter has been the most promising due to its versatile properties and mainly because of the existence of clinically approved magnetite products as an iron supplement and contrast agent for magnetic resonance imaging [9]. However, its potential use in the magnetic targeting of compounds with promising health benefits is still a work in progress.

For decades, natural polymers have played a significant role as biocompatible coatings for IONPs. Chitosan (Ch), in particular, can confer the colloidal stability of the IONPs, provide mechanical integrity to the NP system, and provide functional groups suitable for encapsulating bioactive compounds [10]. Ch has been widely used due to its biodegradable, biocompatible and non-toxic properties. Ch provides a hydrophilic surface coating for stability in physiological environments. The reduced toxicity of IONPs toward healthy tissues can also be attributed to the surface coating [11]. Moreover, the functional groups of Ch can promote colloidal stability, both for the storage of the formulation and when the Ch-coated materials come into contact with biological fluids [12].

Curcumin (CUR) has been widely used as a food supplement due to its reported pharmacologic actions such as antioxidative [13,14,15,16], anti-inflammatory [15,16], anticancer, and antimicrobial agent [17,18,19]. However, its low bioavailability and quick elimination hamper its biomedical applications, forcing an increase in the required amount of CUR to ensure its sufficient intracellular concentration. To overcome the unfavorable physicochemical properties of CUR, we have recently synthesized curcumin diethyl γ-aminobutyrate (CUR-2GE), a carbamate prodrug of CUR with enhanced anti-neuroinflammatory properties [20,21]. However, CUR-2GE is poorly water-soluble, which can be improved by encapsulating it in nanoparticle formulations.

IONPs are widely used as nanomaterials in cancer treatment, especially in drug delivery. The drug-loaded IONPs enter the body by intravenous or arterial injection [22]. Then, they can be directed to the target site by applying an external magnetic field. However, IONPs have many limitations regarding circulation time in the body. They are unstable colloids, easily form aggregates, and are quickly removed by the mononuclear phagocytic system (MPS) [3]. The critical conditions such as size, shape, surface coating, surface charges, and stability must be examined and optimized in synthesis. The optimized conditions of magnetic nanoparticles can prolong their blood circulation time in the body and increase the administration of magnetic nanoparticles to the targeted site [5,6,7,8]. There are no studies on the anticancer potential of the CUR-2GE prodrug. It would be interesting to take advantage of the unique properties of IONPs to target the delivery of CUR-2GE in cancer cells. This was the first study to fabricate a magnetic-responsive delivery system for CUR-2GE using Ch as the coating material and IONPs as the magnetic-responsive core (CUR-2GE-Ch-IONPs) for breast cancer treatment. 

Therefore, this study was undertaken to develop CUR-2GE-Ch-IONPs with desirable attributes for magnetic targeting delivery. We first designed and optimized Ch-IONPs using Box–Behnken design (BBD)-based response surface methodology (RSM). CUR-2GE was then loaded onto the optimized Ch-IONPs. The storage stability and drug release of the optimized CUR-2GE-Ch-IONPs were determined. Their cytotoxicity against MDA-MB-231 breast cancer cells was evaluated.

## 2. Materials and Methods

Chemicals: CUR-2GE was adapted from a previous study [20,21]. The iron (II) chloride tetrahydrate (FeCl_2_∙4H_2_O) was purchased from Thermo Fisher ACROS Organics™ (New Jersey, USA). The iron (III) chloride hexahydrate (FeCl_3_∙6H_2_O), sodium hydroxide, and absolute ethanol were purchased from Carlo-Erba reagents (Val de Reuil, France). Ch (MW = 260 kDa, DD = 90.4%) was supplied by Marine Bio-Resources Co., Ltd. (Samut Sakorn, Thailand).

Cell Culture: Human breast cancer cells (MDA-MB-231) were cultured in Dulbecco’s modified eagle medium (DMEM) supplemented with 10% fetal bovine serum and 1%(*v/v*) penicillin/streptomycin (Gibco, Canada). The cells were cultured at 37 °C in a humidified atmosphere of air: CO_2_ at a ratio of 95:5 (*v/v*).

### 2.1. Fabrication of Ch-IONPs

The Ch-IONPs were prepared by in situ co-precipitation in the aqueous solution of Ch and precipitation with sodium hydroxide (NaOH) using the method previously described [23], with modifications. Briefly, the Ch solution was prepared by dissolving various amounts of Ch powder in an acetic acid solution (0.5% *v/v*, 100 mL) and shaking it at room temperature for 24 h. The Ch solution was then filtered using a 0.45-mm cellulose acetate membrane filter to remove any impurities. Next, 10 mL of the Ch solution was added to 45 mL of ultrapure water and stirred at 1000 rpm for 5 min using a magnetic stirrer (Heidolph, Schwabach, Germany). Then, 10 mL of ferrous chloride (FeCl_2_∙4H_2_O) solution (50 mM) was added dropwise using an automatic syringe pump (Pump 11 Elite, Harvard Apparatus, Holliston, MA, USA) to the aqueous Ch solution and continuously stirred at room temperature for 1 h. Subsequently, 10 mL of ferric chloride (FeCl_3_∙6H_2_O) solution (62.4 mM) was added to the solution and stirred for 1 h. The resulting solution was adjusted with a NaOH solution (4% *w/v*) to obtain solutions with various pH. The solution was heated at different temperatures while continuously stirred for 30 min. The formed Ch-IONPs were precipitated in the solution, separated by a magnet and washed several times with ultrapure water. Finally, the resulting Ch-IONPs were lyophilized (FreeZone 6 Plus, Labconco, Kansas City, MO, USA) at −80 °C for 48 h and stored in a desiccator.

### 2.2. Experimental Designs

#### 2.2.1. Single-Factor Experiments

The single-factor experiments involved three factors, including Ch (MW 75, 260, and 540 kDa), a flow rate of ferrous and ferric chloride solutions (10, 20, and 30 mL/h), and reaction time after adding NaOH (0.5, 1, and 2 h). The responses involved particle size (nm) and saturation magnetization (*M_s_*) (emu/g). 

#### 2.2.2. Optimization Using the BBD-Based RSM

To optimize the fabrication of Ch-IONPs, the BBD-based RSM was investigated and employed with Design-Expert^®^ 13.0.5 (Minneapolis, MN, USA). Table 1 shows the main factors, including pH (*X*_1_), amount of Ch *(X*_2_), and reaction temperature (*X*_3_), as well as three levels of each factor (low (−1), medium (0), or high (+1) values) on the particle size of Ch-IONPs (*Y*). The suitability of the polynomial equation for particle size was analyzed using ANOVA. The response was fitted to linear, second order, and quadratic models and then evaluated in terms of the statistical significance of the coefficients and *R*^2^ values. The validity of the model was confirmed by fabricating the Ch-IONPs under the optimum conditions. The percentage of error, with an acceptable value of <20%, was computed by comparing the observed values with the predicted value of the particle size.

### 2.3. Fabrication of CUR-2GE-Ch-IONPs

CUR-2GE was loaded onto Ch-IONPs (CUR-2GE-Ch-IONPs) using the soaking method as previously described [24] with modifications. Typically, powdered Ch-IONPs (0.3 g) were added into an ethanolic CUR-2GE solution (5 mL, 1 mg/mL) and mixed thoroughly on an orbital shaker (MaxQ 6000, Thermo Fisher Scientific, Waltham, MA, USA) with the speed of 150 rpm at room temperature for 5 h. The resulting CUR-2GE-Ch-IONPs were collected, lyophilized at −80 °C for 48 h, and stored in a desiccator for further experiments.

### 2.4. Characterization

The particle size and zeta potential were characterized using a Zetasizer (Malvern Nano ZS, Malvern Instruments Ltd., Malvern, Worcestershire, UK) based on the dynamic light scattering (DLS) technique. The encapsulation efficiency (EE) and loading capacity (LC) of CUR-2GE–Ch-IONPs were measured using the indirect [24] and direct [25] methods. For the indirect method, CUR-2GE-Ch-IONPs were magnetically separated from the CUR-2GE solution after soaking for 5 h, and the remaining CUR-2GE present in the solution was measured at 401 nm using a UV-Vis spectrophotometer (Cary60, Agilent Technologies, Santa Clara, CA, USA). For the direct method, the dried CUR-2GE-Ch-IONPs were extracted with 1:1 PBS (pH 7.4): absolute ethanol. The CUR-2GE in the extract was determined after magnetical separation using a UV-Vis spectrophotometer (Cary60, Agilent Technologies, Santa Clara, CA, USA). The EE and LC of the CUR-2GE-Ch-IONPs were evaluated using Equations (1) and (2) for the indirect and Equations (3) and (4) for the direct method:

(1) $$\mathrm{EE}\;\left(\%\right)=\frac{\left(W_{i\;}-W_{r}\right)}{W_{i\;}}\;\times \;100$$

(2) $$\mathrm{LC}\;\left(\%\right)=\frac{\left(W_{i\;}-W_{r}\right)}{W_{d\;}}\;\times \;100$$

(3) $$\mathrm{EE}\;\left(\%\right)=\frac{W_{s}}{W_{i\;}}\;\times \;100$$

(4) $$\mathrm{LC}\;\left(\%\right)=\frac{W_{s}}{W_{d\;}}\;\times \;100$$

where *W_i_* is the initial amount of CUR-2GE, *W_r_* is the residual amount of CUR-2GE that remained in the CUR-2GE solution after soaking Ch-IONPs for 5 h, *W_s_* is the amount of CUR-2GE in the extract, and *W_d_* is the dry mass of the CUR-2GE-Ch-IONPs.

The morphology of the CUR-2GE-Ch-IONPs was visualized using TEM (JEM 1400 Plus, JEOL, Tokyo, Japan). The surface functional group of the CUR-2GE, Ch-IONPs, and CUR-2GE-Ch-IONPs were evaluated using an FTIR spectrophotometer (Spectrum One, PERKIN ELMER, Waltham, MA, USA). The surface speciation, oxidation state, and elemental presence of the NPs were characterized using x-ray photoelectron spectroscopy (XPS, Axis ultra DLD, Kratos, Manchester, UK). The thermogravimetric analysis was measured using a thermogravimetric analyzer (TGA; TG 209 F3 Tarsus, Netzsch, Burlington, MA, USA). The samples were processed at a heating rate of 5 °C/min, from room temperature to 800 °C, under a steady flow of nitrogen with a flow rate of 20 mL/min. The crystalline phase of the CUR-2GE, Ch-IONPs, and CUR-2GE-Ch-IONPs were investigated using an X-ray diffractometer (PW3710, Philips, Almelo, The Netherlands). The magnetic properties were measured using an in-house developed vibrating sample magnetometer (VSM; calibrated with a 3 mm diameter Ni sphere model 730908, Lake Shore). 

### 2.5. Storage Stability 

The storage stability of the CUR-2GE-Ch-IONPs was investigated according to the protocol described by Sorasitthiyanukarn et al. [24] with modifications. Briefly, the CUR-2GE-Ch-IONPs were stored at 4 °C and 25 °C. At predetermined times, the physicochemical properties, including particle size, zeta potential, EE, and LC of the CUR-2GE-Ch-IONPs, were measured. 

### 2.6. In Vitro Release of CUR-2GE from CUR-2GE-Ch-IONPs

The release study was performed using a previously reported method [24,26,27] with modifications. In brief, 25 mg of the Ch-IONPs were dispersed in 5 mL (1 mg/mL) of the ethanolic CUR-2GE and shaken at 100 rpm for 5 h. The NPs were separated from the liquid and left to dry for 10 min. The in vitro release of CUR-2GE from the CUR-2GE-Ch-IONPs was carried out at 37 °C using PBS buffers that mimic the blood pH (pH 7.4) and intracellular tumor pH (pH 5.3) as release media. To maintain sink conditions, all release media contain 50% (*v/v*) ethanol and 50% (*v/v*) PBS. The dried CUR-2GE-Ch-IONPs (10 mg) were shaken in 10 mL of the release media at 100 rpm. A total of 200 µL of the supernatant was obtained at each time point (0.25 to 24 h) and immediately replaced with the same volume of the media. The concentration of the released CUR-2GE was measured at 401 nm. All procedures were performed in triplicate. The cumulative release (%) was calculated based on Equation (5):

(5) $$\mathrm{CR}\;\left(\%\right)=\frac{V_{e}\sum_{i=1}^{n–1}C_{n–1}{+V}_{o}C_{n}}{m}\;\times \;100$$

where CR = cumulative amount of CUR-2GE released (%); *V_e_* = sampling volume (mL); *V_o_* = volume of release medium (mL); *C_n_* = concentration of CUR-2GE at the pre-determined time point; and m = total amount of CUR-2GE in CUR-2GE-Ch-IONPs (mg). 

### 2.7. In Vitro Cytotoxicity Studies

The cytotoxicity of the CUR-2GE and CUR-2GE-Ch-IONPs against cancer cell lines was assessed using an MTT assay, following a modified procedure described by Sorasitthiyanukarn et al. [24]. Briefly, MDA-MB-231 cells were seeded at a density of 3 × 10^4^ cells/100 μL/well in 96-well culture plates and incubated overnight at 37 °C with 5% CO_2_ for 24 h. The cells were washed with serum-free complete medium (CM) and incubated with CUR-2GE or CUR-2GE-Ch-IONPs with different CUR-2GE concentrations at 37 °C. The effect of the presence of the permanent magnets on the cytotoxicity results was evaluated by comparing three different exposure times (1, 2, and 4 h) of the NP-treated cells to the permanent magnets. The permanent magnets were placed under each well of the culture plate and incubated at 37 °C for 1 h. After 1 h, the permanent magnets were detached, and the well plate was continuously incubated at 37 °C for 23 h. Similar steps were conducted for the 2 and 4 h exposure time to the permanent magnets. The Ch-IONPs, free CM, and 0.5% (*v/v*) DMSO were used as controls. After a 24 h treatment, the media were removed, and cells were washed with PBS. Then MTT (0.5 mg/mL in PBS) was added to each well and incubated at 37 °C for 2 h. After that, the medium was removed, followed by the addition of 100 µL DMSO to dissolve the formazan crystals. The optical density was measured at 540 nm using a microplate reader (CLARIOstar, BMG Labtech, GmbH, Germany). Cell viability was calculated using Equation (6):

(6) $$\mathrm{Cell}\;\mathrm{viability}\;\left(\%\right)=\frac{N_{t}}{N_{C}}\;\times \;100$$

where *N_t_* and *N_c_* are the optical densities of the treated and untreated (control) cells, respectively.

### 2.8. Live/Dead Cell Staining 

To visualize the live/dead cells, MDA-MB-231 (3 × 104 cells/well) were seeded into a 24-well plate and were treated with CUR-2GE-Ch-IONPs containing various CUR-2GE concentrations (1.33, 5.34, 21.33 μg/mL) with and without 4 h exposure to the permanent magnets. After 24 h incubation, nuclear staining with 10 μM Hoechst33342 and 5 μg/mL PI was performed to detect apoptotic and necrotic cell death in MDA-MB-231 cells, respectively. The bright blue fluorescent signals of Hoechst33342 and the red fluorescent signals of PI were visualized by an inverted fluorescence microscope (Olympus IX51, Olympus Corp., Tokyo, Japan). 

### 2.9. In Vitro Cellular Uptake

In vitro cellular uptake of CUR-2GE and CUR-2GE-Ch-IONPs was visualized by an inverted fluorescence microscope (Olympus IX51 inverted microscope, Tokyo, Japan). The experiments were adapted from previous work with modifications [28]. MDA-MB-231 cells (3 × 10^4^ cells/well) were seeded and incubated overnight to ensure cell attachment. Afterward, the medium was replaced with serum-free DMEM containing the free drug and sample suspensions. The effect of the presence of the permanent magnets on the cellular uptake of the NPs was evaluated by exposing the NP-treated cells to the permanent magnets. The permanent magnets were placed under each well of the culture plate and incubated at 37 °C for 1 h. After 1 h, the permanent magnets were detached, and the cells were washed with PBS. DAPI was added to the cell and incubated at 37 °C for 15 min. Cellular uptake was expressed as the difference between the fluorescence intensity signals from the CUR-2GE-Ch-IONPs and those from CUR-2GE. 

### 2.10. Statistical Analysis

All quantitative data were collected in three replicates and expressed as mean ± standard deviation (SD). The calibration curve was analyzed using simple linear regression and checked for assumptions of linearity, normality, homoscedasticity and outliers. The differences in means between various treatments were determined using ANOVA and Tukey’s multiple comparisons test. Differences were considered significant with a *p*-value < 0.05. The IC_50_ values for the free CUR-2GE and CUR-2GE-Ch-IONPs were determined through a non-linear regression curve fit analysis. All statistical analyses were done using GraphPad^®^ Prism 9.3.0 (San Diego, CA, USA).

## 3. Results and Discussion

### 3.1. Experimental Designs

#### 3.1.1. Single-Factor Experiments

The single-factor experiments involved three factors, including the effects of the molecular weight (MW) of Ch (75, 260, and 540 kDa), a flow rate of ferrous and ferric chloride (10, 20, and 30 mL/h), and the reaction time after adding NaOH (0.5, 1, and 2 h). The responses involved particle size (nm) and saturation magnetization (*M_s_*, emu/g). The single-factor experiments were conducted to identify the optimum material and processing conditions to prepare the Ch-IONPs [29]. The Ch-IONPs with the highest *M_s_* and the smallest size increase the potential for magnetic-assisted delivery to the targeted site [5]. 

**Figure 1:**
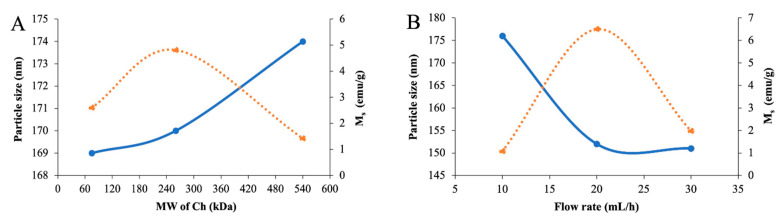

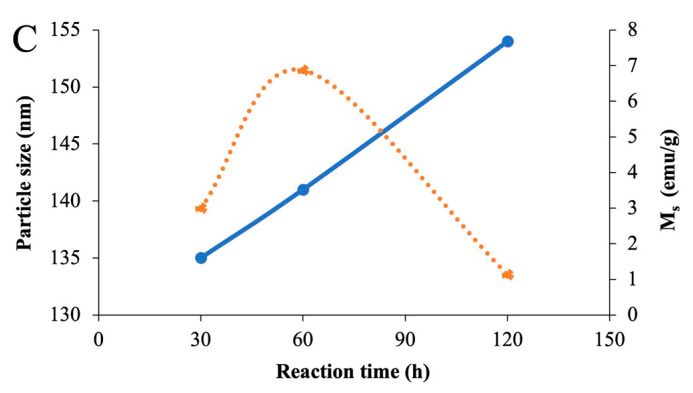
Effect of (**A**) MW of Ch, (**B**) flow rate of Fe precursors, and (**C**) reaction time after adding NaOH on the size (blue) and *M_s_* (red) of the Ch-IONPs.

The MW of Ch is an essential factor that can influence the size and the *M_s_* of the Ch-IONPs. A higher MW of Ch provides a greater density to coat the IONPs, resulting in bigger particle sizes. Consequently, a thicker Ch coating lowers the *M_s_* of the Ch-IONPs. The Ch-IONPs were fabricated by varying the MW of Ch at 75, 260, and 540 kDa to evaluate its effect on particle size and *M_s_* while keeping the flow rate of ferrous and ferric chloride and reaction time after adding NaOH constant at 20 mL/h and 1 h, respectively. As shown in Figure 1A, there was an increasing trend in size from 75, 260, and 540 kDa. Generally, IONPs would range from 5 to 150 nm. The chitosan polymer network provides confined spaces to allow the crystal growth of magnetite, resulting in small particles that are homogeneously distributed. High MW Ch provides more chain segments for interaction with other Ch chains, leading to a more viscous dispersion that provides a thicker coating layer and increases in size. The *M_s_* increased from 75 to 260 kDa, while it decreased when the MW increased from 260 to 540 kDa. Coating the IONPs with chitosan reduces *M_s_* values because of the denser chitosan matrix. Highly viscous media would result in the formation of clusters of magnetic nanoparticles with multiple domains, leading to a decrease in their magnetism. The decrease in *M_s_* can also be attributed to the formation of smaller IONPs using an MW of 260 kDa compared to an MW of 540 kDa. The IONP crystal orientation becomes more disordered with smaller sizes, leading to a decrease in magnetism [30,31,32]. Therefore, 260 kDa was considered the optimal MW in the experiments. 

Flow rate is another critical factor that can influence the formation of Ch-IONPs. In this study, Ch-IONPs were fabricated by varying the flow rate at 10, 20, and 30 mL/h to evaluate its effect on the size and *M_s_* while keeping the MW of Ch at 260 kDa and reaction time after adding NaOH at 1 h. The results in Figure 1B show that size decreased as the flow rate increased from 10 to 30 mL/h. Even though the flow rate was increased, there was no appreciable difference in the size of the Ch-IONPs between 20 and 30 mL/h flow rates. We speculate that at a higher flow rate, the impact of energy is higher, leading to a greater degree of dispersion of the Fe ions with the Ch in a shorter period, and, while the reaction rate is faster, settling, and Brownian movement is not favored, resulting in smaller particles. At a lower flow rate (10 mL/h), the residence time of the Fe ions with the polymeric chitosan chains is increased, allowing more aggregation and settling to occur, resulting in bigger particles. This was in agreement with the findings in the literature, where the dosing rate only moderately affected the particle size. The results also show that *M_s_* increased from 10 to 20 mL/h while decreased from 20 to 30 mL/h. The longest reaction time with the lowest flow rate (10 mL/h) is conducive to oxidation, thus converting the magnetite to another form of IONPs with a reduced *Ms*. The *M_s_* of the product obtained with the highest flow rate was lower than that obtained with 20 mL/h due to the short reaction time of the Fe ions with Ch, resulting in partially embedded Fe ions within the chitosan chain, which may be partially oxidized [33,34]. The results indicated that 20 mL/h was the most favorable flow rate for the fabrication of the Ch-IONPs.

The effect of the reaction time of the Fe ions embedded in the Ch chains with NaOH was considered necessary as it affected the nucleation and growth patterns of the IONPs. The experiment was carried out by varying the reaction time at 0.5, 1, and 2 h while fixing the MW of Ch at 260 kDa and the flow rate at 20 mL/h. Figure 1C shows that the particle size increased when the reaction time was lengthened. The crystal growth of Fe_3_O_4_ starts with the supersaturation of Fe^2+^ and Fe^3+^ ions with the polymer matrix. This is followed by nucleation, which provides tiny crystalline nuclei. The nuclei undergo a growth and aggregation step through the diffusion of the Fe ions on the surface of the nuclei. This is followed by a rearrangement to form single crystalline particles. The chitosan prevents polydispersity and aggregation for the initially formed nuclei. However, as the reaction time increases, the particles continue to grow in all directions. In some cases, uncontrolled growth, or Ostwald ripening, happens wherein bigger particles grow at the expense of smaller ones, thus forming clusters or aggregates. The formation of larger particles or aggregates with an increasing reaction time may be due to the magnetostatic interaction of magnetite particles. The results also show that the *M_s_* increased from 0.5 to 1 h and decreased from 1 to 2 h. The results indicate that the reaction time influenced the formation of the IONPs. We speculate that a longer reaction time led to a complete crystallization of the IONPs, resulting in a higher magnetism. The shortest reaction time would result in an incomplete co-precipitation process, thereby reducing the *M*_s_ [35]. The longest reaction time (2 h) would result in the oxidation of the IONPs to other forms of iron oxide, which reduces the *M_s_* [32,36,37]. These results suggest that a reaction time of 1 h was the optimal condition for fabricating the Ch-IONPs. 

#### 3.1.2. Optimization Using the BBD-Based RSM

Based on the literature review and preliminary experiments, a three-level-three-factor BBD was appropriate to determine the optimum conditions for fabricating the Ch-IONPs. The pH (*X*_1_), amount of Ch (*X*_2_), and reaction temperature (*X*_3_) were the factors selected, while particle size was selected as the response. Particle size was chosen as the response because it is one of the essential factors in determining biodistribution kinetics. A suitable size of the IONPs results in a prolonged blood half-life and increases their chance of accumulation in the tumor site [5]. The experimental matrix and results of the BBD are shown in Table 2. The experiments were carried out randomly using the run order to eliminate positional bias. It can be observed that there were considerable variations in particle size with different fabrication conditions.

Multiple regression analysis showed the second-order regression model (Equation (7)) to predict the particle size for fabricating Ch-IONPs. An analysis of variance (ANOVA) was conducted to determine the coefficients in the regression model that significantly describe the relationship between the factors and responses (Table 3). The *F*-value of the model (35.68) and the associated *p*-value (0.0005) indicate that the factors in the regression model adequately describe the response. The *F*-value (18.37) for the lack of fit and its associated *p*-value were at the borderline cut-off value of 0.05. This phenomenon can be attributed to the insignificant coefficients in the model (*p* > 0.05). Nevertheless, all the coefficients were retained to adequately describe the functional relationships among the factors and the response. Moreover, the regression model has adequate precision (21.2047), indicating the suitability of the model to navigate the design space. Based on the observed effects of the linear coefficients, the amount of Ch (*X*_2_) significantly and positively influenced the size of the fabricated Ch-IONPs. On the other hand, the pH (*X*_1_) negatively influenced the size of the Ch-IONPs whereas the reaction temperature (*X*_3_) did not show a significant effect (Table 3). In addition, the quadratic term coefficient, *X*_1_^2^, was significant, while other coefficients were not significant.

(7) $$\mathrm{Size}=156.33\;-\;20X_{1}+48.00X_{2}\;-\;2.75X_{3}\;-\;6.25X_{1}X_{2}+0.2500X_{1}X_{3}\;\;-\;0.75X_{2}X_{3}+16.71{X_{1}}^{2}+6.71{X_{2}}^{2}\;-\;6.29{X_{3}}^{2}$$

**Figure 2:**
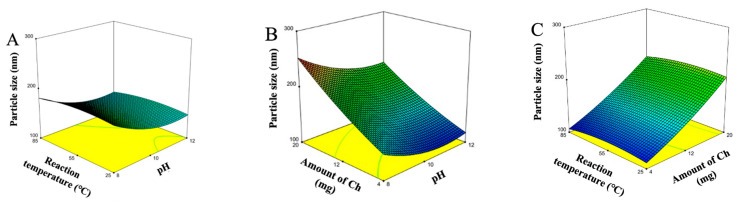
Response surface plots showing the effects of the interaction of (**A**) pH (*X*_1_) and reaction temperature (*X*_3_), (**B**) pH (*X*_1_) and amount of Ch (*X*_2_), and (**C**) amount of Ch (*X*_2_) and reaction temperature (*X*_3_), while keeping the other factor at the zero level (middle level), on the size of Ch-IONPs.

The response surface plots show the three-dimensional (3D) representations of the regression models (Figure 2A–C). These graphical displays are important as they clearly show the effects of the various levels of each factor and the interactions of the two factors on particle size. The plots show the combined effects of two factors at any level while keeping the level of the other factor at zero or mid-level. The quadratic effects of the coefficient, *X*_1_^2^, were displayed by the evident curvature of the 3D plots containing *X*_1_ as a factor. These Figures show that particle size increased initially and then decreased with increasing pH. This confirmed that the optimum pH for the reaction with the precipitating agent (NaOH) was near the zero level. The Figures also show the effects of the reaction temperature with an almost flat line, suggesting that any temperature condition within the experimental testing range could be utilized in fabricating the Ch-IONPs. This signifies that the synthesis reaction could occur at ambient temperature, which will benefit the investigator. Moreover, the results show that increasing the amount of Ch leads to bigger Ch-IONPs. A minimum particle size can be achieved with the minimum amount of Ch. Overall, the quadratic regression model suggested that the amount of Ch (*X*_2_) was the most significant factor that affected the particle size, followed by pH. 

The optimum conditions for fabricating the Ch-IONPs were utilized to achieve their minimum particle size. The optimum conditions were determined as follows: a pH (*X*_1_) of 11, an amount of Ch (*X*_2_) of 4 mg, and a reaction temperature (*X*_3_) of 85 °C. The regression models were validated by performing additional experiments. The optimum conditions were used to fabricate Ch-IONPs with three independent experiments at a desirability of 1.0. The observed particle size was 110.5 ± 6.3, approximately equal to the predicted particle size (104.8 nm). The percentage error was 5.41%, which was < 10%, indicating the predictability of the regression model for the particle size, respectively.

### 3.2. Fabrication of CUR-2GE-Ch-IONPs

**Figure 3:**
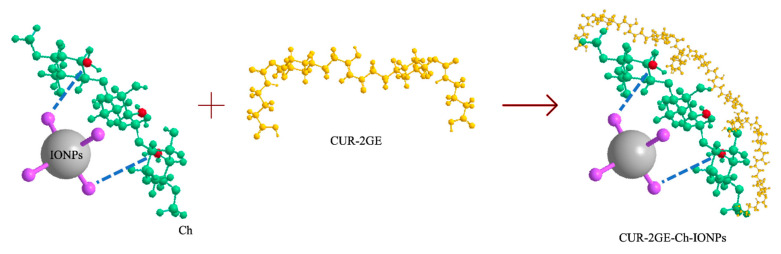
Schematic representation of the fabrication of the CUR-2GE-Ch-IONPs showing the interactions among IONPs, Ch, and CUR-2GE.

The type of nanoformulation produced was a nanosphere wherein Ch, with its several amine groups, forms a complex with the IONPs and CUR-2GE. The amine groups of Ch are responsible for the metal-ion chelation due to the presence of an electron lone pair on the nitrogen atom. The Fe^2+^ and Fe^3+^ components of the IONPs readily interact with the available –NH_2_ groups through ion exchange or complexation reactions [38]. The IONPs form the core of the particle. The Ch network provides not only stability, biodegradability, and non-toxicity but also the surface and functional groups to bind the CUR-2GE molecules [3,4]. CUR-2GE attached non-covalently through hydrophobic interaction and adsorption on the available hydroxyl and amine groups of Ch (Figure 3). 

### 3.3. Characterization

#### 3.3.1. Morphology

**Figure 4:**
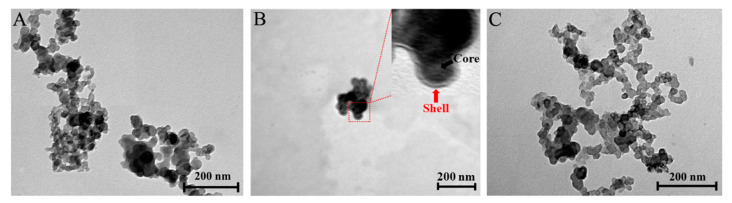
TEM images of (**A**) uncoated IONPs (60,000×), (**B**) Ch-IONPs (40,000×), and (**C**) CUR-2GE-Ch-IONPs (60,000×). The inset in (**B**) shows the core-shell structure of the optimized Ch-IONPs.

The TEM images reveal an agglomeration of the uncoated IONPs (Figure 4). Previous studies reveal a similar phenomenon due to the magnetic dipole–dipole interactions of the particles forming microclusters [39]. The diameter of the uncoated IONPs was approximately 20 nm. The hydrophobic nature of the uncoated IONPs also resulted in their aggregation. The agglomeration process was also the inherent nature of the IONPs to lower their surface energy [40,41]. The inset in Figure 4B reveals the core–shell structure of the optimized Ch-IONPs. The core (IONP) and shell (Ch) were approximately 33 and 3 nm, respectively. The Ch-IONPs may appear as agglomerates due to the processing of the samples during the TEM analysis, as demonstrated by a previous study [39]. The drying process resulted in the shrinking of Ch on the surface of the IONPs and may have built bridges leading to higher interaction with other Ch-IONPs [42].

Figure 4C shows the TEM image of the CUR-2GE-Ch-IONPs. Similarly, the particles appear as aggregates due to the sample preparations. However, the size of the particles was more homogenous compared to the uncoated IONPs. Their size, approximately 27 nm, was lower than the optimized Ch-IONPs, probably due to the effects of the solvent during the loading of CUR-2GE onto the optimized Ch-IONPs. The loading process could have also caused the disappearance of the core–shell structure due to the occupancy of CUR-2GE along the surface and within the polymer shell layer of Ch.

#### 3.3.2. Encapsulation Efficiency and Loading Capacity

Table S1 summarizes the direct and indirect methods for estimating the EE and LC of the CUR-2GE-Ch-IONPs. A comparison was made to reflect the efficiency of the methods in measuring the hydrophobic CUR-2GE molecules physically entrapped in the NPs. The direct method allows a more precise determination of both EE and LC as it involves the use of a solvent to extract and measure the quantity of CUR-2GE directly from the CUR-2GE-Ch-IONPs. However, the t-test revealed insignificant differences (*p* > 0.05) between the indirect and direct methods of extraction for the determination of EE and LC. The low SD also demonstrates the reproducibility of the methods of extraction. Further, the indirect method for estimating EE and LC has often been utilized, as demonstrated by similar studies [43,44,45]. This study demonstrates the efficiency of the indirect method, which is time-saving, in estimating the load of CUR-2GE in the NPs to meet the requirements of Ch-IONP as a carrier system for magnetic targeting delivery applications.

#### 3.3.3. Surface Functional Groups

**Figure 5:**
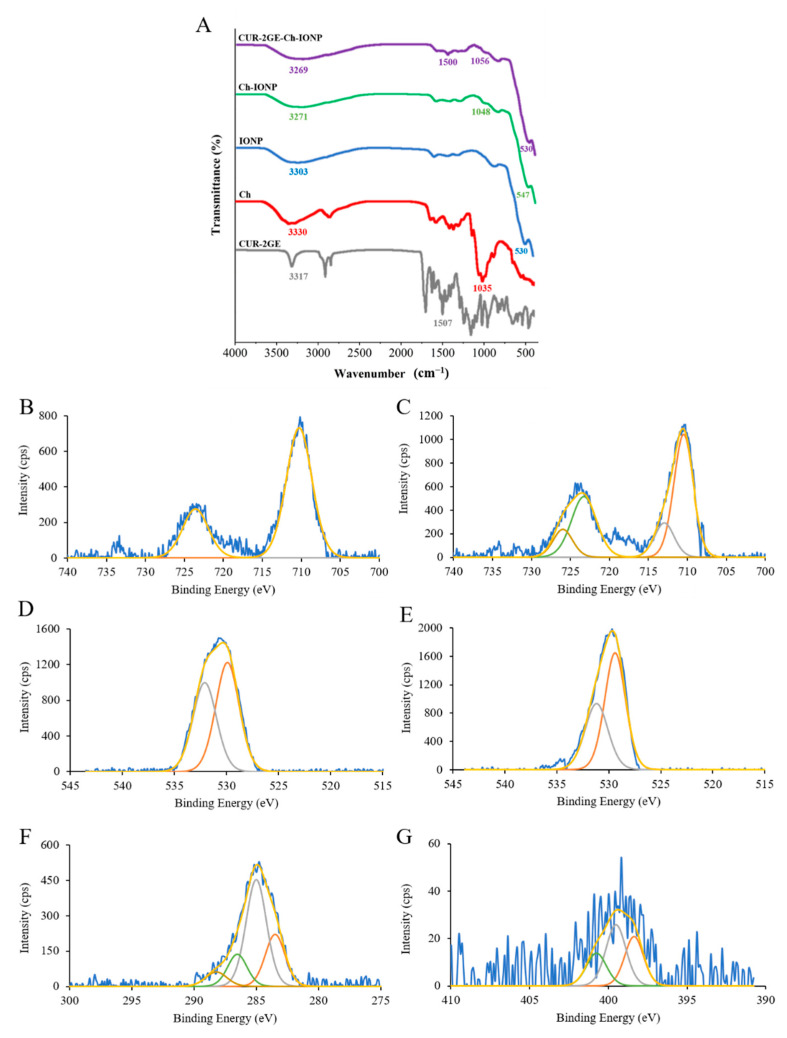
Surface characteristics: (**A**) ATR-FTIR spectra of the samples; XPS spectra of (**B**) Fe 2p spectrum of IONPs; (**C**) Fe 2p spectrum of Ch-IONPs; (**D**) O 1s spectrum of IONPs; and (**E**) O 1s spectrum of Ch-IONPs; (**F**) C 1s spectra of Ch-IONPs; and (**G**) N 1s spectrum of Ch-IONPs.

The surface functional groups of the samples are shown in Figure 5. The characteristic Fe–O vibrations are shown by the bands appearing between 530 and 547 cm^−1^. This confirms the identity of IONP in the uncoated IONPs, Ch-IONPs, and Cur-2GE-Ch-IONPs. In the spectra of the uncoated IONPs, a slightly broad band at 3303 cm^−1^ represents the adsorbed or coordinated water or hydroxyl groups on the surface of iron oxides [46]. This band also slightly shifted to 3271 cm^−1^ and 3269 cm^–1^ in the spectra of Ch-IONPs and CUR-2GE-Ch-IONPs, respectively, due to the weak intermolecular forces of attraction between the adsorbed –OH groups of IONPs and the –OH of Ch. Moreover, this slightly broad peak indicated the N–H stretching in Ch that overlapped with the O–H stretching in IONPs [38]. The presence of Ch in Ch-IONPs and CUR-2GE-Ch-IONPs was confirmed by the appearance of 1048 cm^–1^ and 1056 cm^–1^ bands, representing the C–O in the ether bond in Ch [47]. The peak at 1500 cm^–1^ in CUR-2GE-Ch-IONPs can be attributed to the presence of C=C in the aromatic ring of Cur-2GE. This signal could mean that Cur-2GE was loaded to Ch-IONPs [48].

#### 3.3.4. Chemical State Analysis Using XPS

XPS was also used to confirm the successfully prepared IONPs (Fe_3_O_4_) and Ch coating on their surface. The XPS pattern for the Fe element in the IONPs was shown in Figure 5B, with the binding energies of 710.28 and 723.58 eV attributed to Fe2p3/2 and Fe2p1/2, respectively. These binding energies were also observed in the spectrum of Ch-IONPs (Figure 5C), indicating the ion oxidation state of Fe_3_O_4_ [49]. According to the literature, the peaks for IONPs change to high binding energy and expand due to the presence of Fe^2+(^2p3/2) and Fe^2+^(2p1/2) compared to Fe_2_O_3_. In addition, the satellite peak of Fe_2_O_3_ at the binding energy of 719 eV was much weaker in Fe_3_O_4_ [50]. In our results, as shown in Figure 5B,C, the peak at 719 eV was not observed in the spectrum of IONPs and Ch-IONPs, confirming that the IONPs were Fe_3_O_4_. In addition, the O 1s spectrum (Figure 5E) of Ch-IONPs demonstrated slightly lower binding energy (529.40 and 531.17 eV) compared to that IONPs (529.92 and 532.09 eV) (Figure 5D). The spectrum of XPS also provided the bonding information between IONPs and Ch. The C 1s spectra of Ch-IONPs (Figure 5F) demonstrated major shoulder at 285.00 eV and 286.53 eV associated with C–C and C–O bonds, respectively [51,52], and the binding energy at 399.52 eV (Figure 5G) may ensure that the contribution of high protonated amine (–NH_3_^+^) groups involved in hydrogen bonding owing to N 1s core level [53,54], suggesting that IONPs successfully coated with Ch.

#### 3.3.5. Thermal Analysis 

**Figure 6:**
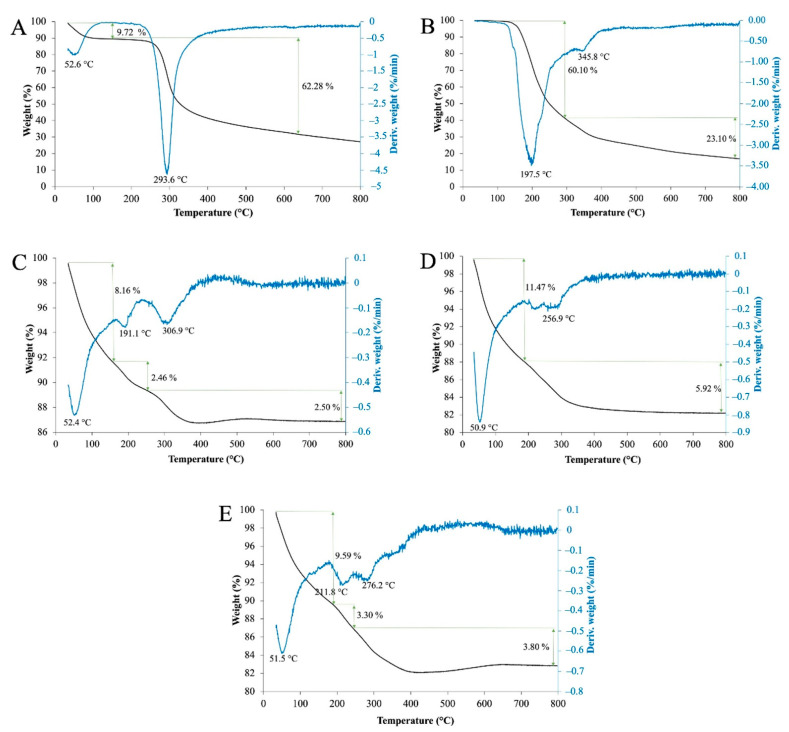
TGA (black) and DTG (blue) curves of (**A**) Ch, (**B**) CUR-2GE, (**C**) uncoated IONPs, (**D**) Ch-IONPs, and (**E**) CUR-2GE-Ch-IONPs.

The thermal behavior of the NPs is illustrated by their TG and DTG curves (Figure 6). As observed in all TG curves, the initial drop indicates the removal of superficially-bonded water or the decomposition of –OH groups. The thermal decomposition of Ch (Figure 6A) is shown by three mass loss events [55]. The first event involves the removal of surface-bonded water molecules, followed by the decomposition of the Ch chains and the degradation of the carbon residues. The thermograms of the uncoated IONPs (Figure 6C) exhibit a mass change at 52.4 °C with a mass loss of 8.16%, which can be attributed to the loss of surface –OH groups, as observed in a similar study [56]. The succeeding mass losses represent the decomposition of the inorganic residues of IONPs. Figure 6D shows the thermal behavior of Ch-IONPs. The mass loss of 11.47% occurring at 50.9 °C signifies water evaporation and surface-bound –OH groups. The decomposition occurring at 256.9 °C with a mass loss of 5.92% indicates the degradation of Ch chains. The residual weight in Figure 6C was higher than in Figure 6D,E due to the higher thermal stability of the uncoated IONPs. The difference in the residual weight corresponds to the degradation of the organic components on the surface of the IONPs. The decomposition temperature in Figure 6E (276.2 °C) was higher than that of Figure 6D (256.9 °C) due to the presence of the thermally stable CUR-2GE. This phenomenon was also observed by comparing Figure 6E with Figure 6B. We can see an increase in decomposition temperature from 197.5 °C (Figure 6B) to 211.8 °C (Figure 6E) due to the hydrogen bonding and hydrophobic interactions of CUR-2GE with the Ch chains.

#### 3.3.6. Crystalline Phase

**Figure 7:**
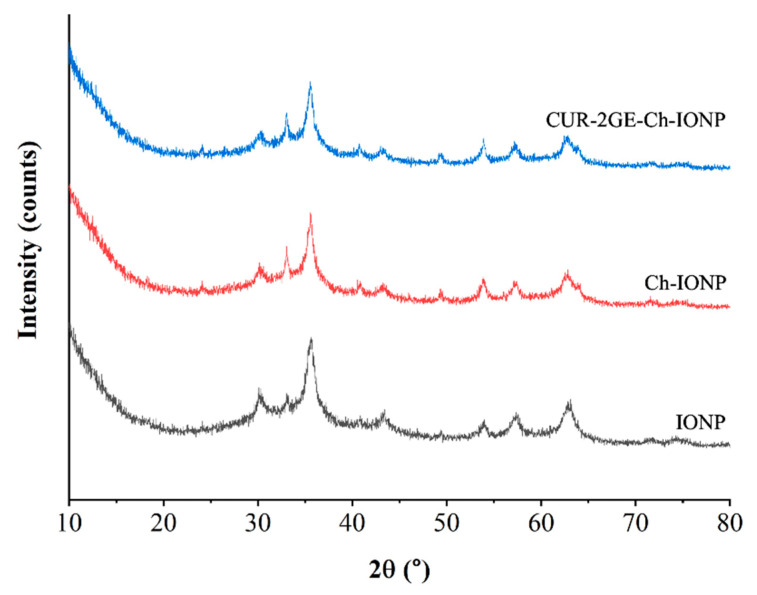
XRD patterns showing the characteristic crystalline nature of the core IONPs in the Ch-IONPs and CUR-2GE-Ch-IONPs.

The XRD patterns are important to reveal the crystalline nature of the IONP core, regardless of the molecules attached. The sharp diffraction peaks that are typical of IONPs are shown in Figure 7. The similarities in the XRD patterns of the uncoated IONPs, Ch-IONPs, and CUR-2GE-Ch-IONPs signify that the coating process and the loading of CUR-2GE preserved the crystalline nature of the IONPs, as there were no changes in the peak positions, as supported by a similar study [56]. The absence of the crystalline peaks of CUR-2GE indicates that it was present in an amorphous form on the Ch chains. Moreover, the XRD patterns reveal that the synthesized IONPs were Fe_3_O_4_ (magnetite). The heat involved in the fabrication process (85 °C) was lower than the temperature range of 220 to 600 °C which would lead to the formation of other forms of IONPs such as hematite (α-Fe_2_O_3_) and maghemite (γ-Fe_2_O_3_), as supported by previous studies [39,57,58].

#### 3.3.7. Magnetic Properties

**Figure 8:**
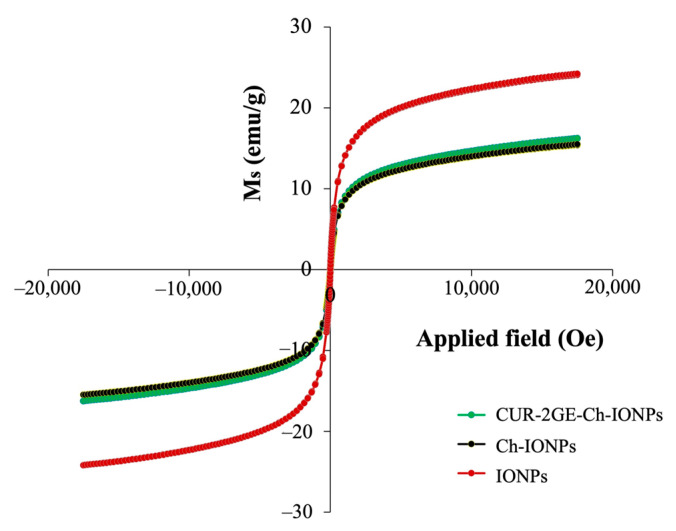
Magnetization behavior of the samples at room temperature.

The magnetic properties of the uncoated IONPs, Ch-IONPs, and CUR-2GE-Ch-IONPs were evaluated using a VSM. As can be seen, Figure 8 indicates the room temperature magnetization curves of the NPs. A comparison of the saturation magnetizations (*M_s_*) of the NPs reveals that the *M_s_* of the uncoated IONPs (24 emu/g) was higher than that of Ch-IONPs (15 emu/g) and CUR-2GE-Ch-IONPs (16 emu/g). These results are in agreement with similar studies, where the coating of the IONPs resulted in a decrease in the *M_s_* due to the addition of non-magnetic components on the surface of the IONPs [59,60]. The IONP’s TEM size (<50 nm) reveals that they are small enough to be superparamagnetic, as evident by their negligible coercivity and remanent magnetization of the IONPs. The superparamagnetic property is essential, as the nanoparticles do not display any magnetization in the absence of a magnetic field, resulting in a stable IONP suspension during the preparation and processing of the IONPs. This property is also desirable as the particles can respond to an external magnetic field and be led to the target tissues in the body, supporting their applications in magnetic targeting delivery [59,61].

### 3.4. Storage Stability Tests

**Figure 9:**
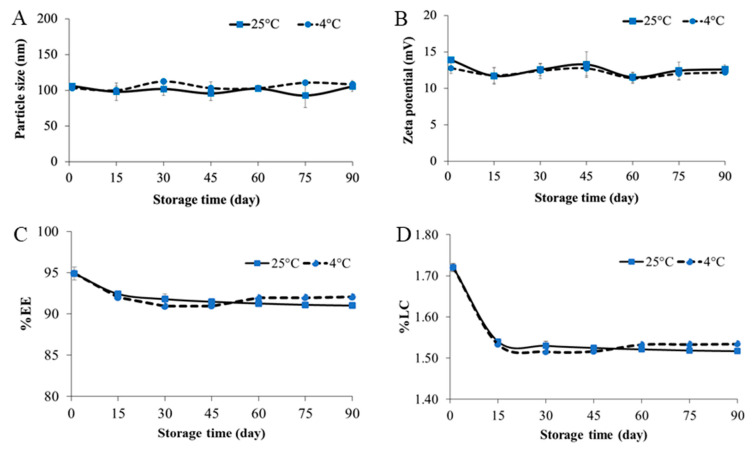
Stability studies showing the particle size (**A**), zeta potential (**B**), retention of CUR-2GE in the CUR-2GE-Ch-IONPs through EE (**C**) and LC (**D**) for up to 90 days of storage at 4 °C and 25 °C.

The storage stability of CUR-2GE-Ch-IONPs in the dried powder form at 25 °C (ambient temperature) and 4 °C (refrigerator) was presented in Figure 9. The results demonstrated that the size and zeta potential of CUR-2GE-Ch-IONPs were not much different during 90 days of storage at 25 °C and 4 °C (Figure 9A,B). In addition, the retention of CUR-2GE in the CUR-2GE-Ch-IONPs through EE (Figure 9C) and LC (Figure 9D) slightly decreased for up to 90 days of storage at 25 °C and 4 °C. Overall, the stability results suggest that the CUR-2GE-Ch-IONPs in the dried powder form can be stored at either 25 °C or 4 °C to maintain their particle size, zeta potential, and retention of CUR-2GE.

### 3.5. Release of CUR-2GE from the CUR-2GE-Ch-IONPs 

**Figure 10:**
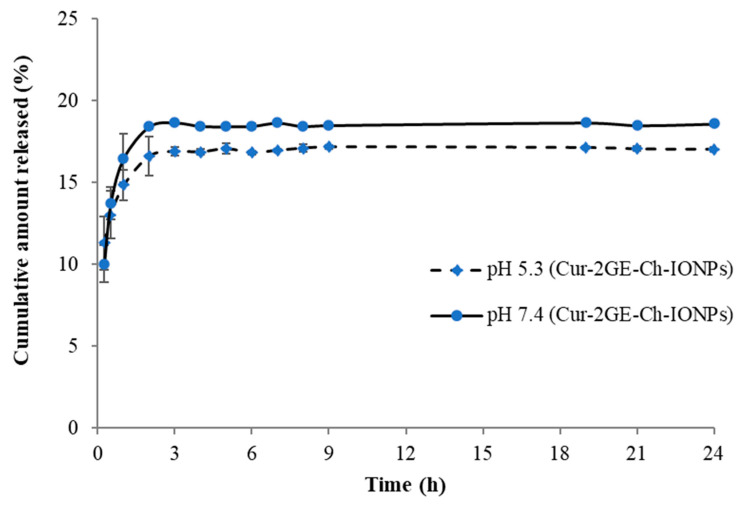
Release profiles of CUR-2GE from the CUR-2GE-Ch-IONPs at pH 5.3 (intratumoral pH) and pH 7.4 (blood pH).

The saturation solubilities of CUR-2GE in PBS (5.3 and 7.4) with 50% ethanol were 0.72 and 0.88 mg/mL, respectively. Figure 10 shows the release profiles of CUR-2GE-Ch-IONPs at pH 5.3 (intratumoral pH) and pH 7.4 (blood pH). These results indicate that Ch-IONPs can control CUR-2GE’s release in each medium. For pH 5.3 and 7.4 media, CUR-2GE exhibited a burst release from the Ch-IONPs during the initial release time points, which can be stable at 4 and 3 h, respectively. This behavior can be attributed to the hydration of the Ch layer and the detachment of the CUR-2GE molecules, which were adsorbed on the NP surface [62]. CUR-2GE was then released continuously until the saturation point. In addition, the release of CUR-2GE at pH 5.3 was less than that at pH 7.4, showing its pH dependency. These results can be attributed to the properties of Ch and are substantiated by similar findings [63]. The release was higher at pH 7.4 due to the deprotonation of the available amine groups in Ch, increasing the pore size of the Ch network and driving the entry of the counterions of the release medium, eventually leading to faster diffusion of the CUR-2GE from the CUR-2GE-Ch-lONPs. The release was lower at pH 5.3 due to the protonation of the amine groups of Ch, leading to the increase in the counterion density followed by repulsion and swelling of the Ch. The swelling of Ch exposes its hydrophobic groups, which tends to slow the migration of the entrapped CUR-2GE to the NP’s surface and release medium due to hydrophobic interactions [64]. These results are essential for predicting the possible release of CUR-2GE into the systemic circulation. Theoretically, the CUR-2GE must be retained in the Ch-IONP carrier for a prolonged period at pH 7.4 to effectively deliver a high quantity of CUR-2GE to the target tumor site. Nevertheless, a sustained-release profile of CUR-2GE can be expected once the CUR-2GE-Ch-lONPs are intracellularly internalized through endocytosis [65]. These results are important for predicting the possible release of CUR-2GE into systemic circulation. To maximize the cytotoxic effects of CUR-2GE on the breast cancer cells, the CUR-2GE-Ch-IONPs should be directly introduced to the tumor to facilitate the accumulation of the particles and release CUR-2GE within the tumor site.

### 3.6. In Vitro Cytotoxicity Studies

**Figure 11:**
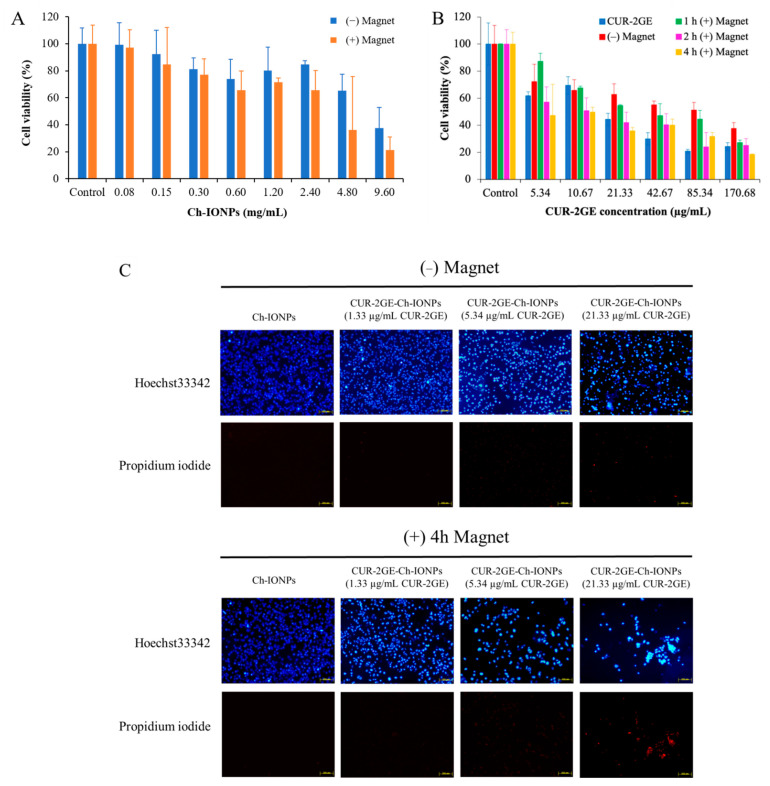
Cell experiments showing the viability of MDA-MB-231 treated with (**A**) Ch-IONPs, (**B**) free CUR-2GE and CUR-2GE-Ch-IONPs at various time points of exposure to the permanent magnets, and (**C**) live/dead cells staining using Hoechst-33342/propidium iodide. The designations (+) and (–) indicate the presence and absence of the permanent magnets, respectively.

The viability of MDA-MB-231 was evaluated using a CUR-2GE concentration range of 5.34 to 170.68 µg/mL (Figure 11B). It can be observed that MDA-MB-231 was sensitive to the effects of CUR-2GE at the minimum concentration tested, with a viability of <70%. A compound or the NPs were considered toxic using a cell viability value of 70% [66]. The CUR-2GE concentration range was also utilized as the basis for the quantities of the CUR-2GE-Ch-IONPs with the equivalent amounts of CUR-2GE. There was a need to investigate the effects of the blank NPs (or Ch-IONPs) on the MDA-MB-231 cells to evaluate the cytotoxic effects of Ch, as its cytotoxicities against cancer cells have been well-documented [67]. The cytotoxic effects of Ch can be attributed to its cationic nature, which may electrostatically interact with the anionic lipid components of the membrane of MDA-MB-231 cells. A concentration of 0.3 mg/mL of Ch-IONPs with magnetic stimulation showed more than 70% viability and was considered non-toxic (Figure 11A). This concentration was subsequently used to dilute the CUR-2GE-Ch-IONP treatment samples.

The cytotoxicity effects of the Ch-IONPs were matched with that of CUR-2GE-Ch-IONPs to investigate whether the cytotoxic effects could be attributed to CUR-2GE molecules or the nanocarriers (Ch-IONPs). Based on Figure 11B, different cytotoxic effects could be observed in the CUR-2GE-Ch-IONPs-treated cells in the presence or absence of the permanent magnets. The time-dependent effects of the magnetic force on the accumulation of magnetic NPs have been previously demonstrated [68]. Various exposure time points to the permanent magnets have been utilized in similar in vitro studies ranging from 0.25 to 4 h [69,70,71,72]. However, these studies have not accounted for the time-dependent cytotoxicity of the magnetic NPs. Furthermore, in vivo magnetic targeting studies in tumor-bearing mice have used permanent magnet exposure of 1 h [44,73,74] or 2 h [75]. The cytotoxic effects of three different exposure time points to the permanent magnets (1 h, 2 h, and 4 h) were then compared. Overall, there was a decreasing trend in the viability of MDA-MB-231, with the highest cytotoxic effects observed with the longest exposure time to the permanent magnets. 

The mean IC_50_ values of the free CUR-2GE and the CUR-2GE-Ch-IONPs were calculated to compare their cytotoxicities toward MDA-MB-231 (Table 4). A one-way ANOVA was used to determine the presence of significant differences among the mean IC_50_ values of the treatments. Tukey’s test was used to compare the multiple mean IC_50_ values simultaneously. The IC_50_ values at differing time points reveal that the cytotoxic effects of the CUR-2GE-Ch-IONPs-treated cells with magnet exposure differed from the CUR-2GE-Ch-IONPs-treated cells in the absence of the permanent magnets. Interestingly, a significant difference (*p* ˂ 0.0001) was observed between the treated cells exposed to permanent magnets for 4 h and the treated cells not exposed to the magnetic field (*p* < 0.0001), demonstrating the successful application of magnetic targeting delivery. The CUR-2GE-Ch-IONPs treatment, with a 4 h magnet exposure time, was ≈ 12 times more potent than the free CUR-2GE. The observed cytotoxic effects as a function of the exposure time to the permanent magnets may be attributed to the nanosized structure, superparamagnetism, and crystallinity of the fabricated CUR-2GE–Ch-IONPs. These properties favorably led to the attraction of the NPs toward the magnetic field that consequently mediates the accumulation of the NPs in the cells [71]. It may also be attributed to the effects of the magnetic field on the release of CUR-2GE, the sustained release of CUR-2GE from the CUR-2GE-Ch-IONPs, and the subsequent apoptotic effects of CUR-2GE. The results of the MTT assay were confirmed by the Hoechst-33342/PI staining (Figure 11C). The 4 h magnet exposure increased the number of apoptotic cells compared to those without magnetic field exposure. This study demonstrates that the presence of the permanent magnets significantly enhanced the cytotoxicity of the CUR-2GE-Ch-IONPs toward MDA-MB-231, a cell line that represents triple-negative breast cancer and is known to be resistant to various chemotherapeutic agents. However, a magnetic field-assisted delivery in murine models will provide more predictive behavior of the CUR-2GE–Ch-IONPs in humans. In most cases, the permanent magnets are placed on the surface of the skin near the shallow tumor to precisely deliver the magnetically responsive NPs to the diseased tissue. These approaches are necessary to better understand the effectiveness of magnetic targeting delivery in a clinical setting [73].

### 3.7. In Vitro Cellular Uptake

**Figure 12:**
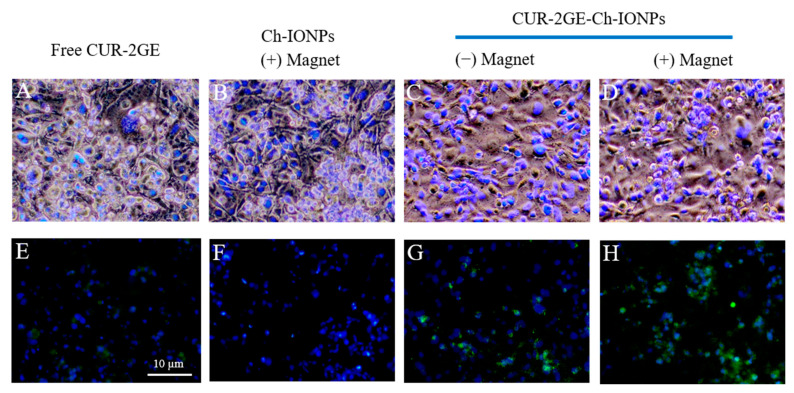
Cellular uptake showing the microscopic images of MDA-MB-231 after incubation with (**A**,**E**) free CUR-2GE, (**B**,**F**) Ch-IONPs, (**C**,**G**) CUR-2GE-Ch-IONPs in the absence of the permanent magnets, and (**D**,**H**) CUR-2GE-Ch-IONPs in the presence of the permanent magnets.

The cellular uptake assay was conducted to evaluate the effect of the magnetic field on the internalization of the CUR-2GE-Ch-IONPs by tracking the fluorescent CUR-2GE molecules using an inverted fluorescence microscope (Figure 12). Figure 12A–D represents the merged images of the brightfield and DAPI to show the nuclei and cytoplasm of the cells. Figure 12E to Figure 12H denote the merged images of CUR-2GE (green color) and DAPI (blue color-stained nuclei) to define the localization of CUR-2GE into the cytoplasm of the cells. However, the cellular uptake experiments had some limitations, such as the use of 1 h instead of 4 h for the exposure time with the permanent magnets to observe the possibility of internalization of the CUR-2GE-Ch-IONPs. We observed the presence of the green fluorescence signals within the cells with 1 h incubation time, possibly due to the viable cell morphology, hence facilitating CUR-2GE uptake. The difficulty in observing the green fluorescence signals at 4 h may be attributed to the loss of cell viability.

There were sparse green fluorescence intensity signals from the free CUR-2GE due to its hydrophobic nature and possible aggregation within the extracellular spaces. The absence of green fluorescence signals with Ch-IONPs signifies that the green fluorescence signals were from the CUR-2GE. The occurrence of the green fluorescence signals of CUR-2GE from the CUR-2GE-Ch-IONPs in the absence of the permanent magnets can be attributed to the collective effects of the nanosized particles and the cationic nature of Ch in facilitating endocytosis. The green fluorescence intensity was remarkably higher in the presence of the permanent magnets due to the localizing and targeting effects of the magnetic force toward the IONP core.

**Figure 13:**
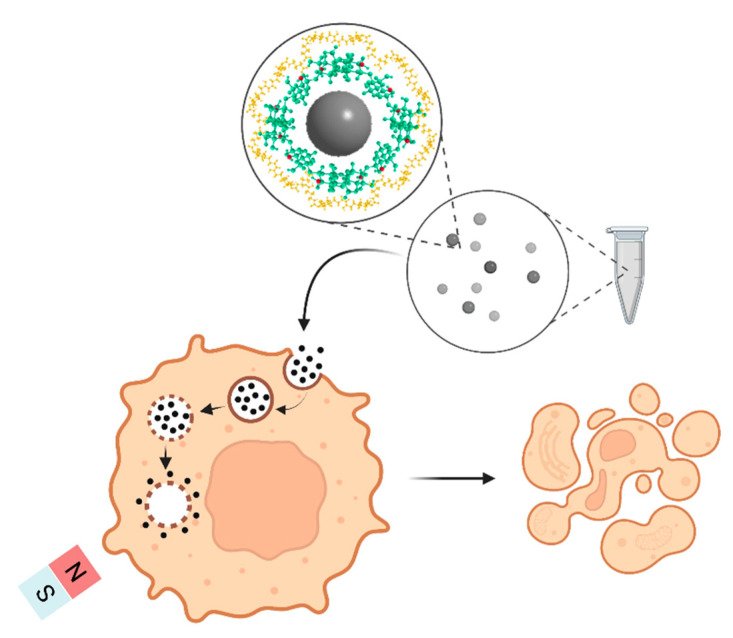
Schematic representation of the cellular uptake and cytotoxicity studies of the CUR-2GE-Ch-IONPs in MDA-MB-231 in the presence of the permanent magnet.

A pictorial representation of the processes leading to the uptake of CUR-2GE-Ch-IONPs, the cytoplasmic release of CUR-2GE, and the cytotoxic effects of the internalized CUR-2GE is depicted in Figure 13. In summary, the endocytic process is facilitated by the nanoscale structure of the CUR-2GE-Ch-IONPs and the magnetic force of the permanent magnets. The cationic nature of Ch imposes an imbalance on the osmotic pressure within the cytoplasm, leading to the endosomal release of the CUR-2GE-Ch-IONPs. The endosomal release triggers the detachment of CUR-2GE from the nanocarrier within the cytoplasm. These phenomena signify the impact of magnetic fields on the internalization of the magnetic-responsive CUR-2GE-Ch-IONPs in MDA-MB-231 and provide evidence of their potential for further development and evaluation using preclinical models.

## 4. Conclusions

This work demonstrated a magnetic-assisted delivery of CUR-2GE in the triple-negative breast cancer cell MDA-MB-231. The magnetically assisted delivery of CUR-2GE in the triple-negative breast cancer cells MDA-MB-231 was demonstrated in this study. The IONPs were deposited in Ch using an in situ co-precipitating process. The fabrication of Ch-IONPs, as the carriers of CUR-2GE, was optimized using the BBD-based RSM. The particle size was < 150 nm. The quantity of Ch and the reaction pH had the highest impact on the size of Ch-IONPs. The incorporation of CUR-2GE occurred through non-specific adsorption, hydrogen bonding, and hydrophobic interaction with the available –NH_2_ and –OH groups and the backbone of chitosan. Incorporating Ch conferred good thermal stability, storage stability, and sustained-release properties. The presence of the permanent magnets significantly enhanced the uptake and cytotoxicity of CUR-2GE against MDA-MB-231, suggesting the great potential of the fabricated CUR-2GE-Ch-IONPs as vehicles for the magnetic-guided delivery of CUR-2GE to breast cancer.

## Figures and Tables

**Table 1:** Variables used in Box–Behnken design.

Variables		Level	
	Low (−1)	Medium (0)	High (+1)
Factors			
*X*_1_ = pH	8	10	12
*X*_2_ = Amount of Ch (mg)	4	12	20
*X*_3_ = Reaction temperature (°C)	25	55	85
Response		Constraint	
*Y* = Particle size (nm)		Minimum	

**Table 2:** Experimental variables and results in the Box–Behnken design (*n* = 3).

Std Order		Factors		Particle Size (nm)
	pH	Amount of Ch (mg)	Reaction Temperature (°C)	
1	8	4	55	148 ± 6
2	12	4	55	111 ± 7
3	8	20	55	261 ± 9
4	12	20	55	199 ± 6
5	8	12	25	181 ± 3
6	12	12	25	150 ± 22
7	8	12	85	183 ± 2
8	12	12	85	153 ± 18
9	10	4	25	117 ± 6
10	10	20	25	210 ± 25
11	10	4	85	105 ± 9
12	10	20	85	195 ± 10
13	10	12	55	154 ± 25
14	10	12	55	156 ± 33
15	10	12	55	159 ± 26

**Table 3:** ANOVA results for evaluating the RSM model for particle size.

Source	Sum of Squares	df	Mean Square	*F*-Value	*p*-Value	
Model	23,228.07	9	2580.90	35.68	0.0005	significant
*X*_1_: pH	3200.00	1	3200.00	44.24	0.0012	
*X*_2_: Amount of Ch	18,432.00	1	18,432.00	254.82	<0.0001	
*X*_3_: Reaction temperature	60.50	1	60.50	0.8364	0.4024	
*X*_1_ *X*_2_	156.25	1	156.25	2.16	0.2016	
*X*_1_ *X*_3_	0.2500	1	0.2500	0.0035	0.9554	
*X*_2_ *X*_3_	2.25	1	2.25	0.0311	0.8669	
*X*_1_²	1030.78	1	1030.78	14.25	0.0130	
*X*_2_ *²*	166.16	1	166.16	2.30	0.1900	
*X*_3_ *²*	146.16	1	146.16	2.02	0.2144	
Residual	361.67	5	72.33			
Lack of Fit	349.00	3	116.33	18.37	0.0521	not significant
Pure Error	12.67	2	6.33			
Cor Total	23,589.73	14				

**Table 4:** Mean IC_50_ values of the free CUR-2GE and CUR-2GE-Ch-IONPs against MDA-MB-231.

Treatment			IC_50_ (µg/mL)	
Free CUR-2GE			17.01 ± 1.63 *	
	(–) magnet		(+) magnet	
		1 h	2 h	4 h
CUR-2GE-Ch-IONPs	65.62 ± 3.46 **	40.92 ± 2.51	11.41 ± 7.25	1.47 ± 1.04

Notes: * *p*-value (0.0150) and ** *p*-value (<0.0001) compared to 4 h (+) magnet.

## Data Availability

Not applicable.

## Supplementary Materials

The following supporting information can be downloaded at: https://www.mdpi.com/article/10.3390/polym14245563/s1, Table S1: Summary of the EE and LC based on the indirect and direct methods of extracting CUR-2GE from the CUR-2GE-Ch-IONPs.
