# Supplementary material for: Fabrication of Curcumin Diethyl γ-Aminobutyrate-Loaded Chitosan-Coated Magnetic Nanocarriers for Improvement of Cytotoxicity against Breast Cancer Cells
Source: Polymers (Basel). 2022 Dec 19;14(24):5563. doi: 10.3390/polym14245563 (PMC9785553; doi:10.3390/polym14245563)
Supplement: Supplementary file 1 [file polymers-14-05563-s001.zip › polymers-2070848-supplementary.pdf]

Supplementary information

Table S1. Summary of the EE and LC based on the indirect and direct methods of extracting CUR-2GE from the CUR-2GE-Ch-IONPs.

Parameter	Indirect method	Direct method	*p-value*
EE	95.5 ± 0.9	96.1 ± 2.2	0.68
LC	1.64 ± 0.02	1.60 ± 0.04	0.10
